# Understandings of depression among community members and primary healthcare attendees in rural Ethiopia: A qualitative study

**DOI:** 10.1177/13634615211064367

**Published:** 2021-12-23

**Authors:** Bethlehem Tekola, Rosie Mayston, Tigist Eshetu, Rahel Birhane, Barkot Milkias, Charlotte Hanlon, Abebaw Fekadu

**Affiliations:** 1 4616 King's College London; 2 201340 Addis Ababa University; 3 Brighton and Sussex Medical School

**Keywords:** depression, Ethiopia, explanatory model, primary care, qualitative method, symptoms

## Abstract

Available evidence in Africa suggests that the prevalence of depression in primary care settings is high but it often goes unrecognized. In this study, we explored how depression is conceptualized and communicated among community members and primary care attendees diagnosed with depression in rural Ethiopia with the view to informing the development of interventions to improve detection. We conducted individual interviews with purposively selected primary care attendees with depression (n = 28; 16 females and 12 males) and focus group discussions (FGDs) with males, females, and priests (n = 21) selected based on their knowledge of their community. Data were analyzed using thematic analysis. None of the community members identified depression as a mental illness. They considered depressive symptoms presented in a vignette as part of a normal reaction to the stresses of life. They considered medical intervention only when the woman's condition in the vignette deteriorated and “affected her mind.” In contrast, participants with depression talked about their condition as illness. Symptoms spontaneously reported by these participants only partially matched symptoms listed in the current diagnostic criteria for depressive disorders. In all participants’ accounts, spiritual explanations and traditional healing were prominent. The severity of symptoms mediates the decision to seek medical help. Improved detection may require an understanding of local conceptualizations in order to negotiate an intervention that is acceptable to affected people.

## Introduction

The World Health Organization estimates that depression affects more than 300 million people globally, which amounts to 4.4% of the world's population (WHO, 2017). Depression is described as the second leading cause of years of life lived with disability (Vos et al., 2012) and a major contributor to disability-adjusted life years (DALYs) (Vos et al., 2017). Those who receive a diagnosis of depression have increased mortality risk by all causes and by suicide (Walker et al., 2015). This is found to be the case across both high- (Gilman et al., 2017) and low-income countries (Fekadu et al., 2015). Depression is commonly comorbid with other chronic diseases such as diabetes or tuberculosis and can worsen associated health outcomes (Ambaw et al., 2018).

In Africa, available evidence suggests that the prevalence of depression is high in primary care settings and often goes unrecognized (Fekadu et al., 2017). Although depression is treatable, treatment is often unavailable, especially in low- and middle-income countries (LMICs) (Patel et al., 2016; Thornicroft et al., 2017). To address this treatment gap in LMICs, the WHO has recommended task-shifted care provided at the primary care level by non-specialists as a key strategy (Patel, 2017; WHO, 2016). However, it has been suggested that the low rates of detection of depression in primary care settings in LMICs make effective treatment of depression difficult (Fekadu et al., 2020; Rathod et al., 2018).

It is now widely recognized that there are cultural variations in the experience, symptomatic expression, clinical presentation, and expected treatment of depression (Kirmayer, 2001; Kirmayer & Bhugra, 2009). Thus, a better understanding of the construct of depression, how it is perceived and reported, and how these processes are shaped by broader cultural factors may contribute to improving detection of depression in primary care settings in LMICs (Andrew et al., 2012; Fekadu et al., 2017; Mayston et al., 2020).

A growing number of qualitative studies have explored explanatory models of depression in different settings in Africa to ensure that detection and treatment are culturally appropriate and relevant to the local context (for a comprehensive review, see Mayston et al., 2020). Explanatory models (EMs) are defined as notions about “nature, name, cause, expected course, and desired treatment for an episode” of illness (Kleinman et al., 1986, p. 84). Behind the concept of explanatory models is the recognition that the experiences and expressions of illness are embedded in the individual's social and cultural context (Kleinman, 1980, 2004). In studies of explanatory models from Africa, EMs have been assessed among lay community members (e.g., Familiar et al., 2013), people living with HIV (e.g., Andersen et al., 2015), primary care attendees with (e.g., Okello et al., 2007) and without a diagnosis of depression (e.g., Irankunda and Heatherington, 2017), and among traditional healers (e.g., Sorsdahl et al., 2010).

In this study, building on this growing qualitative literature, we aimed to explore how depression is conceptualized and communicated among community members and primary care attendees diagnosed with depression in rural Ethiopia with the view to informing the development of interventions to improve detection. In doing so, our study adds to a small number of in-depth qualitative studies on the phenomenology and explanatory models of depression from Africa. This is important particularly in light of a recent qualitative review of literature which highlighted a lack of research carried out among non-Western societies that could improve our global understanding of depression (Haroz et al., 2017), which is mainly based on studies of Western societies. Another recent review of qualitative literature indicated that even within the limited number of studies from Africa, participant samples are dominated by women and the levels of description of participants’ experiences presented varied across studies (Mayston et al., 2020, p. 11).

## Methods

### Setting

The study was conducted in primary healthcare facilities in Sodo district, in the Gurage Zone of the Southern Nations, Nationalities and Peoples’ region (SNNPR) of Ethiopia. SNNPR is one of the largest and most ethnically diverse regions of Ethiopia. The Sodo district is located about 100 km from the capital city, Addis Ababa. It has a population of 170,000, with about 90% living in rural areas. The district was selected for our study because it is the site of a global mental health initiative—the Programme for Improving Mental Health Care (PRIME) (Lund et al., 2012)—which gave us the opportunity to explore the experience of depression in the context of implementing a new mental health service in primary care. This also helped us to develop rapport and trust with participants and to strengthen our understanding of the local sociocultural context.

### Design

The study was conducted as part of the Improving Detection of Depression in Sub-Saharan Africa (IDEAS) project, led by Prof. Abebaw Fekadu (African Research Leader scheme, Medical Research Council, UK). The aim of IDEAS is to develop interventions to improve detection of depression in primary care settings in rural Ethiopia. This involves three steps. First, understanding how depression is conceptualized in rural Ethiopia and how this might influence detection (the current study fits here). Also incorporated in the first step is understanding how primary care providers recognize depression and what factors may moderate the process of detection. Second, based on findings from the first step, developing an intervention package for improving detection. Third, testing the intervention package to see if it is locally acceptable and feasible to deliver and if it can be replicated widely.

A qualitative research design was used for this study. Specifically, we conducted FGDs with community members to gain data on their conceptualization of depression including local terms used. FGDs were also valuable in terms of understanding how group members collectively constructed local meanings, practices, and expressions (Willig, 2013) relevant to our study. We also conducted individual interviews with people diagnosed with depression to elicit their explanatory models. Individual interviewers are best suited to collecting rich and detailed data about participants’ perspectives and experiences (Braun & Clarke, 2013; Willig, 2013).

### Recruitment and sample description

FGDs were conducted with community members composed of males, females, and Orthodox priests (n = 21; seven females and 14 males). The community members were judged to be knowledgeable about issues that concern the community by members of the community advisory board, which was established seven years ago by another mental health research project (Lund et al., 2012) following a series of consultations with the community and the district administration. The board meets twice a year with the project leaders to discuss the ongoing implementation of mental healthcare in the district, and this meeting has also continued under IDEAS. Three FGDs (one comprising only women, one only men, and one only priests) consisting of seven members each were formed. The priests’ group included all men, as priesthood is generally reserved for men in Ethiopia. We set up homogenous focus groups with the assumption that participants who share a common frame of reference such as sex feel more comfortable sharing their views (Morgan et al., 1998), particularly if the discussion involves sensitive issues (Liamputtong, 2011).

Semi-structured individual interviews were conducted with purposively selected primary healthcare (PHC) attendees who had been diagnosed with depression by primary healthcare workers who had been trained in the World Health Organization mental health Gap Action Programme intervention guide (n = 16 women, n = 12 men). During the selection process, we included people who had dropped out of treatment as well as those who remained in care. The interviews were conducted 6–12 months after the diagnosis of depression and the participants had started treatment, which included provision of basic psychosocial care (addressing social stressors) by PHC and antidepressants, if indicated.

The demographic characteristics of the study participants are outlined in Table 1.

**Table 1.** Demographic characteristics of study participants (N = 50).

Characteristics		Participants with depression (individual interviews) n = 29	Community participants (FGDs) (n = 21)
**Gender**	Male	12	14
	Female	16	7
**Age**	18–35 years	11	5
	36–50 years	11	10
	51–65 years	6	6
**Educational level**	No formal education	15	1
	Primary education	8	11
	Secondary education	4	7
	Post-secondary education	1	2
**Marital status**	Single	8	0
	Married	20	21

### Data collection

Focus group discussions (FGDs) were conducted in Amharic (the official working language of Ethiopia) by the last author (lead facilitator) and the third and fourth authors (assistants) at a health facility within the Sodo district. In the FGDs, we used an open-ended approach to explore participants’ conceptualizations. Specifically, FGDs generally started with broad questions about health problems that concern the community, followed by a discussion of mental health conditions in the community and symptoms of mental health conditions. Then, a case vignette describing a woman with symptoms of depression was read to participants. The case vignette was constructed by the last author based on a real-life experience of a woman diagnosed with depression in Ethiopia. The name and other identifying information were changed or removed to protect the woman's confidentiality. The symptoms in the vignette were consistent with the criteria for depression in the Diagnostic and Statistical Manual of Mental Disorders (DSM-5) (American Psychiatric Association, 2013). This was followed by open-ended questions asking community members to conceptualize the problem described in the case vignette and to answer questions such as whether they consider the problem as illness or not. Further questions focused on possible causes and sources of help-seeking and treatment based on Kleinman's (1980) open-ended questions. A similar technique was previously used in studies conducted in Kampala, Uganda (Okello & Ekblad, 2006), in Iran (Dejman, 2010), and recently in South Africa (den Hertog et al., 2021). FGDs lasted an average of 1 h and 28 min.

Individual interviews were conducted in Amharic by the last author and the third and fourth authors at a health facility. With the aim of further developing the interview guide and facilitating analysis, we listened to the first set of interviews before conducting further interviews. The interview guide for individual interviews was also developed based on Kleinman's (1980) open-ended questions. Participants were asked about the symptoms of their illness, its causes, effects, and any treatment received. All interviews were audiotaped and lasted 40–70 min.

### Data management and analysis

Data were analyzed using thematic analysis. Our analysis was guided by existing theories and conceptual frameworks related to explanatory models of illness (Kleinman, 1980). Thus, in our analysis we used a ‘top-down’ or deductive approach to thematic analysis (Braun & Clarke, 2006).

The analysis process involved three main stages. During the first stage, immediately after data collection, the tapes were transcribed verbatim in Amharic. Each interview and FGD transcript was compared with the audiotape to ensure quality. The third and fourth authors, who conducted the individual interviews and assisted in FGDs, and who are bilingual in Amharic and English, conducted the initial analysis of the data. They read and re-read transcripts to further familiarize themselves with the data and generated preliminary codes using OpenCode 4.0 (qualitative data analysis software, University of Umeå, Sweden). During the second stage, the first author who is also bilingual in Amharic and English familiarized herself with the data by repeatedly listening to audio-recorded interviews whilst reading transcripts. Then, she reviewed preliminary codes generated by third and fourth authors in light of the analytical ideas she noted down during the data familiarization process. In the next phase, informed by community members’ explanatory models of illness to further make sense of the data, the first author refined preliminary codes and selected codes that are relevant to the study's research questions. She then combined codes that conceptually go together and grouped all data extracts relevant to each of the combined codes. NVivo 11 was used to assist with this process.

In the third stage, the first author met with the last and second authors to gain their input regarding whether the combined codes fit well with the collected data and were relevant to the study's research questions. During our analysis process, we gave special attention to local (Amharic) words used to explain symptoms and illnesses. Thus, in our findings, whenever necessary, we included Amharic words used along with their English translation.

### Ethical considerations

The study was approved by the scientific committee of the Department of Psychiatry and the Institutional Review Board of the College of Health Sciences of Addis Ababa University (IRB ref: 007/18/PS). All interviews were conducted after obtaining written informed consent from participants.

### Researchers’ reflexivity

The research process (including research design, data collection, and analysis) was inevitably influenced by our perspectives (Finlay & Gough, 2008). The IDEAS project was motivated by concerns about the need for interventions to improve detection of depression. Thus, it is effectively a global mental health project with a biomedical starting point. The last author, who led the research and was also involved in conducting FGDs and individual interviews, has a medical background and has conducted mental health research at the study site for many years. Although he came to the research with a recognition that culture plays a major role in understanding the construct of depression, inevitably his position as a mental health researcher working in a psychiatric hospital may have shaped the research process, including what participants told us (or did not tell us) about their experiences. Although we began all our interviews and FGDs by highlighting to participants the importance of their perspectives to our study and that there are no wrong or right answers, it is possible that their responses might have been influenced by our previous mental health research in the study site and the last author's medical background. It is also possible that participants with a diagnosis of depression might have presented what they thought we would like to hear by interpreting their experiences in light of the biomedical model or by not telling us their experiences that do not fit this model. This is similar to what is referred as the “looping effect” (Kirmayer et al., 2017), which involves “the ways in which people tend to modify their behavior (intentionally or without awareness) as a result of being recruited into certain social categories” (p. 166). The first author who led the analysis and writing process has an anthropology background and worked with qualitative approaches to research which emphasize the context-bound nature of knowledge. This background informs her analysis and the process of writing about participants’ experiences, during which she paid close attention to the local context and detailed description.

## Findings

### Conceptualizations of mental illness

In discussing health problems that concern the community in general, participants in all FGDs emphasized illnesses that are associated with a lack of clean water (specifically typhoid and typhus), diabetes, and high blood pressure. Only one participant from the women FGD and priest FGD each talked about mental illness (የአይምሮ ህመም; ye’āyimiro himemi) unprompted. When asked to specifically talk about mental illness, many of the FGD participants talked about mental illness in relation to addiction (chewing Khat, a local stimulant, and use of alcohol by men), evil spirits, developmental disabilities, and seizure disorders. Four participants talked about distress (ጭንቀት; ch’inik’eti) caused by lack of money, thinking too much, or losing someone they loved because of death. Asked about the symptoms of mental illnesses generally, many of the participants talked about ‘not knowing what one speaks’ / saying meaningless things, unpredictable and aggressive behavior, wandering around, sleeping rough, and lack of personal hygiene. Participants indicated that people with such types of symptoms are often called mad, mentally ill, and mentally retarded.

Often, spiritual explanations such as intergenerational curse or being cursed for doing something bad were mentioned by participants when more than one person was affected by mental illnesses in a family. In talking about mental illness, participants in the priest FGD differentiated between problems that are suitable for holy water treatment (spiritual treatment) and those that are not:

We can say that we know the illnesses that are suited for holy water. Those with mental illness, even if we provide them with service for one day or two days, there is a difference between an evil spirit and a mental illness. Those with mental illness even if they showered in the holy water for one day or two days, what we advised them is to go to a health center for example to Amanual [hospital] in Addis Ababa … if it is a mental illness, we do not want them to stay for long in the holy water place because these are two different things. (Priest FGD no. 1)

Asked about how they can tell whether a person is possessed by an evil spirit or has a mental illness, the priests explained that when showered in the holy water or prayed upon, those who are possessed by an evil spirit will shout and talk (the spirit will say it is ready to go out of the host) and they get cured, while those with mental illness say nothing and the illness does not get better. All except one priest said that there is no difference between the two in terms of symptoms, specifying that mental and spiritual causes can only be distinguished based on whether or not the afflicted person talks when showered with holy water or prayed upon.

### Conceptualizations of a case of a woman with depression

Asked about how they conceptualized the condition of the woman in the vignette (see Appendix A), all except four FGD participants who suggested spirits and spells perceived the symptoms of depression described in the vignette, such as disliking talking to people and neighbors, as a normal reaction to the dissolution of a marriage. Although the facilitator mentioned several times that the separation from her husband had happened nearly eight years before the onset of the symptoms, the separation of the woman in the vignette from her husband (and her resulting loneliness) was the most commonly discussed possible cause of her condition among FGD participants. Only two participants from the priest FGD and one participant from the men and women FGDs each discussed spirits and spells as a possible reason for the woman's condition.

For me the cause of this is: first when God created humans, He made us have partners or marriage partners. I guess the cause could be separation … because the woman is separated from her husband, she would feel lonely … she may get worried about what her future looks like [without a marriage partner] … The cause, as far as I am concerned, is a feeling of loneliness which brought about too many thoughts. (Priest FGD no. 4)

… I can, for example, talk about what has happened to me. My wife and I quarreled and were separated for a certain period. She stayed with the children and I moved out of our home. Because of the separation, at night even if I went to bed, my mind could not sleep. I felt like I was sleeping but my mind would not stop thinking. I did not know what was going on, only my family would tell me in the morning that I was making a sound of pain. My mother would ask me whether I am feeling pain inside. It is only the fact that I was separated from my family that bothered me. I did not have any other problem; I work and earn [money] and spend properly. But the issue of my family was always in my mind. Now, the problem with this woman [the woman in the vignette] is not another thing other than there is a pain inside her because of what has happened to her family. (Male FGD no. 1)

All participants except those who suggested spirits and spells as an explanation perceived the condition described in the vignette as a social problem and not an illness. A participant in the female FGD (no. 2), for example, explained: “raising children on your own, living by yourself can bring about hopelessness. Apart from that I would not see this as an illness.” But participants who suggested social explanations also highlighted that the problem faced by the woman in the vignette can lead to an illness. A participant from the male FGD (no. 1), for example, indicated that separation can rob people of “peace of mind” which can result in mental illness.

Participants in the priest FGD pointed out that when faced with challenges such as the one described in the case vignette, the first point of intervention should be getting advice and support from family and friends: “… it is necessary to have someone who supports you emotionally, gives you advice and educates you. When there is such kind of person the woman can return to her old self” (Priest FGD no. 3). However, if the woman's condition gets worse and affects her mind participants suggested medical intervention. The accounts of participants in the priest FGD suggest that getting worse means not getting better even after receiving advice and support from others. If the woman's condition does not improve after receiving social support, one of the priests suggested taking her to a health center:

The life which used to be shared with her husband can be difficult for her when she is alone … she needs support, she needs a helper, she needs advice. If her problem persists and gets worse, she should seek help from professionals who could help her … she should go to the health centre (Priest FGD no. 6)

Even some of the participants who suggested social problems (i.e., the separation of the woman from her husband) as a possible reason for her condition gave spiritual explanation for her separation, suggesting that social and spiritual explanations are intertwined in their accounts. One of the participants from the male FGD (no. 5) argued that separation cannot be a reason for the woman's condition, pointing out that after she separated from her husband, the woman managed to live happily for eight years. He said it is possible that an evil spirit sent to her by other people is controlling her mind, making her isolate herself from people, and further causing her to dislike people and be unhappy. He argued that an evil spirit can even lead to madness.

Asked about how other people such as their neighbors would conceptualize the condition of the woman in the vignette, all participants from the female FGD pointed out that many people would associate the woman's condition with a spell that has been cast onto her by her husband. A similar conceptualization was also given by one of the participants from the priest FGD:

The one who is saying you are going to die could be a spirit. This spirit may have captured her and caused her to be gloomy and lose her appetite. There are different kinds of illnesses and spirits are many. (Priest FGD no. 7)

Going to a holy water place or other traditional healing places were suggested when the cause of the woman's condition was believed to be spirits and spells. Asked about whether they have come across people who show similar symptoms (such as people who isolate themselves from social life) as the woman described in the case vignette, a participant from the male FGD (no. 5) talked about a man who isolates himself from his family and the community. Asked about the cause of the man's problem, the participant said it could be the influence of a spirit as it is difficult to find any other explanation: “there is nothing that he does not have. He did not lose his business. He was doing ok.”

### Explanatory models of people with depression attending PHC and symptom presentations

Participants who visited the health center and were identified as having depression talked about their condition as illness. Asked about what they and other community members name their illness, most participants said it is called distress (ጭንቀት ነው) and distress of the mind (የአንጎል ጭንቀት ነው; የአእምሮ ጭንቀት; ye’ānigoli ch’inik’eti newi; ye’ā’imiro ch’inik’eti).

Asked about what they thought caused their illness, many of the female participants traced their illness to the time they experienced control and violence from men such as their husbands and siblings. Explaining how her illness began, a married woman with four children said she went to Dubai to work and earn money for the family, leaving her husband and children at home. When she came back home from Dubai and discovered that her husband had spent all the money she had sent home with another women. She recalled:

I became very angry, crying day and night, I became distressed, then, crying and crying. When people came, I cried, they asked “How is your effort [What is the result of your work in Dubai]?”; those who went with me [to Dubai] changed, they built a house. When I saw their change and the house [they built] my head was affected. I became sick, I lost my appetite, I got distressed, my whole body. It was like losing my consciousness, I did not know what it was. Then my son took me to *Butajira* hospital. (Patient 9)

Another married woman with eight children indicated how her husband's control of their possessions (which included cattle and grains) was linked to her illness:

… he [husband] often makes me angry, he spent all my possessions and controlled them. I was like a person in prison [who could not do anything]. I just waited for him [at home doing nothing]. How is it, how do I go out, how do I get back [home], how do I work, whether it is a marketplace or a place for grinding mill. When I questioned this and got angry, I thought that it is better to die than this …. Instead of being angry living in this house I said it is better to die and drank a medicine [to kill myself]. (Patient 7)

Failing in life despite hard work, not being able to provide for their family, and comparing oneself with others who are believed to have a good life were mentioned as illness explanations by many of the male participants:

I am a father. I have eight children; at home, together with my wife and myself, we are 10. When I think of this illness, what it is, I work hard, I am a hard worker, but I am unable to succeed; when I buy cattle for farming, I only use them for a year, they die, and my life is not like other people. Some people even when they are not serious about work, and not working, they live a good life. I ask God, why me? I get angry … (Patient 3)

Interviewer: What do you think is the cause of this problem?

Participant: This distress could come because a human being always wants to live a happy life in this earth. One compares himself with others. When I have a life less than the other [person] I say: “Why don't I live like this? What prevented me [from living like this]? I work, I work, why doesn't my life change?” Saying that [by thinking like that] I distressed my mind. (Patient 8)

The expectation that married men need to provide for their family and the negative consequence of not being able to meet this expectation are illustrated in the following quotes:

The distress, I have mentioned it earlier to some extent, since I am a human being, my desire to get, I always desire to live a better life. I always think to improve my life. Rather than my children suffer I would prefer for me to suffer and when my children eat good food and dress like other children, I consider it as something I have done, and I become happy. When I am unable to do that, it affects me inside a lot. (Patient 8)

Elsewhere in his interview, the participant explained that although his wife is hardworking and brought income for the family while he was sick, the fact that he was unable to fulfil the traditional gender role (i.e., men work outside the house and bring in income while women stay at home and are responsible for raising children and doing household chores) has affected him:

… and when she came home after working, while I was sitting at home like a woman, when she came home after working outside like a man, I question the food I eat, I say this to myself, but I don't speak this out, there is this feeling, it is still there.

Explaining how their illness began, some of the female participants mentioned cultural practices during the bereavement period and giving birth. A female participant, for example, associated her distress with the death of her parents and brother as well as the practice of not putting butter or oil on her body and scalp during the bereavement period which resulted in dry skin and scalp (see Appendix B).

### Symptom presentation

The most common symptoms spontaneously reported by participants with depression included problems related to sleep (such as difficulty falling asleep, awakening at night, difficulty going back to sleep, sleepiness in the day), tiredness, being distressed, disliking noise, an urge to shout and disappear, or an urge to disappear / to get out of the house and move. Table 2 presents the most common symptoms spontaneously reported by participants with depression, local (Amharic) words used, and whether these symptoms are included in the current Diagnostic and Statistical Manual of Mental Disorders (DSM-5) and the International Classification of Diseases (ICD).

**Table 2.** Most common symptoms spontaneously reported by participants with depression.

Symptom dimensions	Spontaneously reported symptoms	Local (Amharic) words used	Included in DSM-5/ICD-10 criteria?
Somatic	Problems related to sleep (difficulty falling asleep, awakened at night, difficulty going back to sleep, sleepy in day)	እንቅልፍ አልተኛም (inik’ilifi ālitenyami); ስተኛ እንደ ተጠራ ሰው አባኖ ያስነሳኝ ነበር (sitenya inide tet’era sewi ābano yasinesanyi neberi); የተወሰነ እተኛና ቶሎ እነቃለሁ (yetewesene itenyana tolo inek’alehu); አንዴ ከነቃሁ ደግሞ በጣም ይቆያል (ānidē kenek’ahu degimo bet’ami yik’oyali); ቀን እንቅልፍ እንቅልፍ ይለኛል (k’eni inik’ilifi inik’ilifi yilenyali)	Yes
	Tiredness	ድካም ድካም ይለኛል (dikami dikami yilenyali)	Yes
	Feeling dizzy	ወደታች ወደ ላይ ነበር እሚያረገኝ (wedetachi wede layi neberi imīyaregenyi); ያዞረኝ ነበር (yazorenyi neberi)	No
	Headache	ያመኝ ነበር ጭንቅላቴን (yamenyi neberi ch’inik’ilatēni); የዕራስ ምታት (ye’irasi mitati)	No
Emotional	Being distressed	ይጨንቀኛል (yich’enik’enyali); ያጨናንቀኛል (yach’enanik’enyali); ጭንቅ ይለኛል (ch’inik’i yilenyali)	Yes
	Loss (complete loss) of interest in work; loss of initiative to work	ስራ መስራት ጭራሽ አልፈልገውም ነበር (sira mesirati ch’irashi ālifeligewimi neberi)	Yes
	Disliking noise	ጫጫታ አይወድልኝም (ch’ach’ata āyiwedilinyimi)	No
	An urge to shout and disappear (run out of the house); an urge to shout; an urge to disappear / to get out of the house and move	ጮኽሽ ጥፊ ጥፊ ይለኝ ነበር (ch’oẖishi t’ifī t’ifī yilenyi neberi); ጩሂ ጪሂ የሚለኝ (ch’uhī ch’īhī yemīlenyi); ጥፊ ጥፊ ይለኝ ነበር (t’ifī t’ifī yilenyi neberi); ብረሪ ብረሪ፣ ሂጂ ሂጂ ይለኛል (birerī birerī፣ hījī hījī yilenyali)	No
Cognitive/affective	Suicidal thoughts	መኪና ውስጥ ግቢ ግቢ ይለኝ ነበር (mekīna wisit’i gibī gibī yilenyi neberi); እራሴን አጥፋ አጥፋ የሚል ውስጤ ላይ ስሜት ነበረው (irasēni āt’ifa āt’ifa yemīli wisit’ē layi simēti neberewi)	Yes
	Being tearful / wanting to cry	በቃ አለቅሳለሁ (bek’a ālek’isalehu); አልቅስ አልቅስ ይለኛል (ālik’isi ālik’isi yilenyali); አልቅሽ አልቅሽ ይለኛል (ālik’ishi ālik’ishi yilenyali)	Yes (but not emphasized)

Asked about the reasons for visiting a PHC center and the symptoms of their illness, interviewed participants mentioned both somatic (such as tiredness) and psychological (such as lack of interest in work) symptoms. Disliking noise, which included noises coming from children talking and playing, people talking, and a radio playing, was mentioned by both female and male participants, although it was mentioned mainly by female participants: “My mind did not like it when people make noises … noises if children talk to each other, that made me very angry. I felt it. Eh, I even did not like to enter a room which had noises” (Patient 1, female);

Even now, I don't like noise eh, even now if the children turn on the radio, I become distressed. When the children make lots of noise, when they play, I feel it eh I don't like it … my head gets disturbed eh if there is lots of noise from people and the children … if I sit down in a place which has noise, my head becomes distressed eh it gets disturbed. If the children also play, inside me there is no peace, my head gets disturbed. (Patient 2, female)

One of the female participants even indicated that her husband brought her to the health center after she broke their radio because she could not stand the noise. She explained that she also disliked the noise coming from people talking and even the noise coming from the chicken: “when the chicken outside makes noises, inside my head a noise would come” (Patient 16). Participants who mentioned disliking noise often talked about being distressed and their head/mind getting disturbed and becoming angry when hearing noise. Noise was presented as something not good for one's peace of mind and emotional state.

An urge to shout and disappear was almost exclusively reported by female participants. All the women who mentioned this also said they had suicidal thoughts such as stepping in front of a moving car and drinking a medicine to die by suicide. Most of them also mentioned having sleeping problems, such as difficulty falling asleep and awakening at night. They also talked about being trapped/imprisoned.

## Discussion

Drawing on the theoretical framework of explanatory models (Kleinman, 1980), this study explored the ways in which depression is conceptualized and communicated among community members and primary care attendees diagnosed with depression in rural Ethiopia. Our findings suggest that there were differences as well as similarities in the conceptualizations of the two groups. While the former might be explained by the influence of engagement with services (context) on explanatory models (Mayston et al., 2020), the latter may point to their shared socio-cultural context. Many of the community members in FGDs did not talk about mental health conditions unprompted. When mental health conditions were discussed, depression was not the focus. Problems raised were those related to addiction (chewing Khat and use of alcohol by men), evil spirits, intellectual disability, and seizure disorder. The symptoms of these identified problems were perceived to be severe/disruptive, having a strong impact on day-to-day living, such as incomprehensibility of speech, lack of predictability, aggression, roaming, and poor personal hygiene. Likewise, in a study from a rural setting in Ethiopia, Alem et al. (1999, p. 44) found that “one has to display behaviour that attracts public attention to be recognized as mentally ill,” and our findings suggest that depression is not naturally placed in this category (see also Deribew and Tamirat, 2005; Monteiro and Balogun, 2014 in Ethiopia and Patel, 1995 in other sub-Saharan African countries).

When participants were presented with a vignette of a woman living with depression, her symptoms and experiences were recognizable, but primarily categorized as a social problem. Because of separation from her husband and the resulting loneliness, many participants argued, the woman in the vignette was having too many thoughts, getting worried about her future, and losing hope. It was expected that if this woman received sufficient social support, the problem would be alleviated over time. The problem was not considered serious compared to “mental illness,” but FGD participants emphasized that the problem faced by the woman in the vignette could become worse and affect her mind. If this happened, they suggested seeking medical intervention, specifically going to a health center. Participants’ accounts suggest that “getting worse” and “affecting her mind” were assessed on the basis of whether or not the woman went back to her usual self/functioning (such as socializing with her neighbors) after receiving suitable social support. Participants only began to think about other possible causes (apart from social problems) and treatment (apart from social support) after the woman's symptoms became severe and their suggested intervention did not work (see also Mayston et al., 2020, in sub-Saharan Africa, and Roberts et al., 2020, in India).

In our study, participants with a diagnosis of depression labelled their problems as an illness but still perceived the cause as primarily social in nature. Their conception was probably influenced by how health workers had explained their problem to them and/or by the biomedical context of the interview. Mayston et al. (2020) also reported that people already engaged with the health system for another illness such as HIV were more likely to describe their depression in biomedical terms, compared to participants without a diagnosis.

Consistent with other studies from Africa (see Mayston et al., 2020) and elsewhere (e.g., Roberts et al., 2020 in India), in our study social explanations (such as relationship problems) were prioritized by both community members and participants with a diagnosis of depression. However, both groups retained uncertainty as to the etiology of the problem. Some community members in FGDs indicated that some of the symptoms of the woman described in the case vignette could be a result of spiritual influence or a spell that has been cast onto the woman by her husband or other people. Some of the participants’ accounts suggest that spiritual explanations were given when there was no other obvious explanation (particularly economic explanation) for a problem or when more than one person was affected by a mental health condition in a family. When spiritual explanations were given, participants suggested going to holy water and/or other traditional healing places for treatment. Similarly, a study in southwest Ethiopia found that one of the most common explanations given for the causes of major depressive disorder, schizophrenia, and other psychotic disorders was spiritual possession and that traditional healers were the first place where help was sought (Girma & Tesfaye, 2011). Alem et al. (1999, p. 45) also indicated that in their study “no informant thought spirit possession could be helped with modern medicine.”

Some of the participants with a diagnosis of depression gave more than one explanation for their illness concurrently. For example, one of the women who said her illness is related to spirit attack (*ልክፍት ‘likift’*) also said the death of her parents and sibling had impacted her mind and worsened her illness. Some of the participants’ seemingly economic explanations also had a spiritual tone. For example, some of the participants associated their perceived inability to succeed in life despite hard work with a spiritual influence, e.g., being cursed or being bewitched or an evil spell being sent to them by others. This was also true regarding the explanations given to the condition of the woman in the case vignette by community members; some perceived the woman's condition as a result of a dissolution of a marriage as well as a result of spiritual influence or spell. This lends support to the findings of Williams and Healy (2001, p. 473) who concluded that “individuals may hold a variety of explanations simultaneously or may move rapidly from one belief to another.” Kleinman (2013) also emphasized the dynamic nature of people's beliefs and conceptualizations and warned against seeing an explanatory model as “a single, unchanging, material thing in the patient's record” (p. 3).

In terms of gender differences, in their presentation of symptoms female participants with depression in our study described disliking noise more often than male participants. An urge to shout and disappear (run away) was exclusively reported by female participants. Although not included in the DSM-5, noise intolerance is described in the literature as part of a Western conceptualization of depression (e.g., Stansfeld, 1992). Previous studies have also discussed noise intolerance or sensitivity to noises in relation to depression in non-Western contexts (e.g., Halbreich et al., 2007 in Tunisia). However, the relative prominence given to noise intolerance in our study is relevant. Our findings on gender difference contrast with what Andrew et al. (2012) found in India. Among PHC attendees suffering from common mental disorders, they found few gender differences in terms of presentation of symptoms. However, in line with their findings on explanatory models, in our study, married female participants related their distress with their lack of power and autonomy and the resulting mistreatment and exploitation they were experiencing from their spouses, whereas male participants often talked about the financial and economic difficulties they faced as a cause of their distress. Previous studies in rural Ethiopia also indicated that among married women, the experience of physical violence, emotional violence, and spouse control were factors independently associated with depression episodes (Deyessa et al., 2009; Gossaye et al., 2003).

In our study, both female and male participants with depression spontaneously reported a combination of somatic and psychological symptoms. The most common psychological symptoms reported were being distressed and lack of interest in work. Consistent with the results of a recent qualitative review (Haroz et al., 2017), our findings suggest that participants living with depression experienced many of the core symptoms found in the current diagnostic criteria for depressive disorders. However, four of the most commonly mentioned symptoms by our participants are not part of current diagnostic criteria, specifically: disliking noise, feeling dizzy, headaches, and an urge to shout and move away to ‘disappear.’ Tearfulness was also commonly mentioned by our participants although not emphasized in the DSM-5 criteria. Haroz and colleagues also found that some important symptoms identified from qualitative studies in particular contexts were missing from international diagnostic criteria. Sadness was not often mentioned spontaneously by our participants. However, in some of the male participants’ explanations about their distress, the importance of being successful, having a good life and being happy was highlighted. This contrasts with the assumption that pursuing happiness is exclusively a Western cultural value. This also goes against the explanation given by some researchers of the low detection and lower prevalence of depression reported in non-Western settings, arguing that depression rates are higher in countries that place an emphasis on happiness (e.g., De Vaus et al., 2018).

### Implications

Our findings have implications for research and clinical practice on depression. Our participants differentiated between mild cases which they perceived as a problem but not an illness and more severe cases which they considered as an illness, particularly when the problem disrupted their day-to-day life. They noted that mild cases can be resolved or improved with social support while more severe cases require going to a health center, suggesting that their perceptions of the severity of symptoms influenced their decision to seek medical help or not. It is therefore essential that health workers understand patients’ perceptions of their symptoms and problems to negotiate an intervention that is acceptable and effective for them (Kirmayer, 2001). This requires building sufficient rapport and trust with the affected person. An understanding of culturally specific symptoms and local terminologies used by people who have depression, not just those contained in the standard diagnostic criteria, is also likely to be helpful in terms of improving detection. Additionally, given that spiritual explanations and traditional healing were prominent in participants’ accounts, a system of care for depression that considers traditional approaches and local resources is likely to be more effective.

### Limitations

We acknowledge the following methodological limitations. Mental health research has been conducted at our study site for several years and our participants may be more familiar with mental health issues than people in other parts of Ethiopia. We interviewed PHC attendees who had been identified as having depression at a health facility by PHC workers trained in a Western construct of depression. All interviews were conducted by mental health researchers and the location of the interviews in the health center may have biased participants’ responses towards the biomedical model. Their responses might have been different if we had selected a location that is part of their local world, e.g., their home. In the FGDs, we only used a case vignette of a woman. It is possible that the discussions might have been different if we had used a case vignette of a man or both. In addition, the fact that the woman in the vignette was separated from her husband might have influenced the participants’ responses. A vignette which describes other social stressors might have produced different responses. However, our focus on a social problem in the vignette is justified by the fact that social explanations of depression are very widespread in the literature from sub-Sahara Africa (see Mayston et al., 2020) and depression also commonly occurs in the context of social adversity.

## Conclusion

By combining FGDs and in-depth individual interviews, this study provided a rich account of the conceptualizations of depression among community members and people with a diagnosis of depression. To the best of our knowledge, this kind of work has not been previously carried out in Ethiopia. It is also one of the very few studies on depression from Africa that engaged multi-disciplinary researchers and attempted to understand the conceptualization and expression of depression from a predominantly rural setting.

The findings of this study suggest that participants conceptualized depression as different from ‘madness’ and perceived the severity of symptoms as important for the decision to seek medical intervention. Many participants remained uncertain and flexible about illness/problem explanation. In the process of trying to work out why something had happened to them or others, many of them seemed to resort to spiritual explanation, particularly when there was no other obvious explanation for the problem. Concerns about life were gendered, and not being able to deal with these was part of the explanation for depression.

## Appendix A

### Case vignette

Tadelech is a 45-year-old woman. She is a farmer. She has two sons aged 16 and 10. She got divorced from her husband eight years ago when her youngest son was two years old. After the divorce, she continued to live her life without any difficulty. She kept running the family's farm. She continued to have income and did not face any financial difficulty. She also continued to have a good relationship with her neighbors. But one day she suddenly started to have a sad feeling. She became weepy. She lost her appetite. She lost weight. She lost concentration. She started to be shy. She started to dislike talking to her neighbors and other people. She lost interest in work. Her situation got worse and she became hopeless. She started to fear that she is going to die and decided to live as a monk in Debre Libanos monastery, leaving her sons behind.

## Appendix B

### Categories and examples of perceived causes reported by participants with depression

[Table table3-13634615211064367]

**Appendix B table3-13634615211064367:**

Category	Perceived cause	Explanation and/or exemplary quote
Exploitation and mistreatment because of lack of power and autonomy	Conflict with husband Conflict with oldest son Conflict with older brothers	A participant whose husband used to drink alcohol heavily explained that her distress was related to the way her husband treated her and their children: “when my husband talks to me bitterly, I get distressed … when he comes home after drinking [alcohol] when he talks to the children while they are working in a negative way, I get disturbed” (Patient 27). A married female participant explained that her distress began after her oldest son who often quarreled with her and her other children threatened to kill them with a blade (Patient 15). Explaining how her illness began, a young unmarried woman noted that after the death of her parents when she was very young (she did not remember them), she took the responsibility of looking after her two older brothers who were often ill. She explained how she was overburdened with work and responsibilities at home. But after they got married, she recalled, her brothers abandoned her in favor of their wives. She said one of her brothers even took her property (such as cattle and grains). She explained that in rural areas women are not expected to own property as when they get married their property will be taken by their husbands. All these circumstances, she noted, affected her health: “I got infected (ተለከፍኩ) [my mind is affected]. At school they wondered what happened to me and used to pour or sprinkle water over my head [when I fainted] … then I started to take medicine [to kill myself]” (Patient 13).
Economic/financial difficulty	Failing in life despite hard work Not being able to provide for their family	A man who is married with five children explained that his illness started when he started to think too much after he lost his money and other possessions due to burglary. He associated the burglary with being bewitched or an evil spell directed at him in the form of dead animals such as a dead snake head being thrown on his shop before the burglary: Interviewer: How did the illness begin? Can you explain that to me? Participant: When it [the illness] started my shop was burgled. I went to the [police] station. I was thinking too much and could not sleep. I spent the night thinking without any sleep. Interviewer: What were you thinking? Participant: About my life, about the things I lost in the burglary … Interviewer: What things did you lose during the burglary? Participant: All my possessions. In fact, after I distributed a letter, the Kebele [local administration] has helped me to some extent. My illness started after this [incident]. (Patient 22) A married woman with seven children (Patient 23) who was responsible for bringing income for the family as her husband did not work because of illness (she said his illness is related to an evil spell put on him (በቃ ህመሙ ማለት ነው የሆነ የሰው እጅ ሳይሆን አይቀርም)) explained that her “mind is affected” (አንጎሌ የተነካው) after her cattle which are the source of income for the family kept dying.
Other familial problems	After loss of parents; financial difficulty which led to inability to continue education which then resulted in distress Distress after discontinuing education because of physical violence from a father	Asked how his distress started, a young male participant (Patient 26) associated his distress with discontinuing his education for lack of money. He explained that after the death of his parents (he doesn't know his mother and remembered little about his father), he started to live with his grandmother and supported himself by working as a shoeshine boy to go to school. But he was forced to discontinue his education after he completed 10th grade (when he was about to join preparatory school): Participant: You need money for food and rent. My grandmother's economic capacity is very limited. Interviewer: So, you could not join preparatory school? Participant: It is not possible … there is house rent, there is food expense. Secondly, if you don't study it [the education at preparatory school] is impossible (Patient 26). A young male participant (Patient 6) traced his distress to the time he quarreled with his father (አዎ በፊት ግን ጭንቅ ምናምን በፊት ከአባቴ ተጣልቼ ጆሮዬን መቶኝ ነበር). He explained that his distress started five years ago after his father hit him on the ear, which resulted in him discontinuing his education.
Supernatural explanations	Being possessed by an evil spirit (likift) God brings it (dependent on God's will)	Participant: Before I went to Amanuel [hospital]. They say it is kind of spirit attack [ልክፍት likift] and I was sitting. Interviewer: What did they do saying likift? Participant: They did not do anything. They took me to the holy water place. I was pregnant [ቅሪት ነበርኩኝ] at that time. They say I cannot drink [the holy water]. I just went there and then went back home. It did not help me at all (Patient 16, female). • When she was asked about how her illness began, a female participant said her illness started after she gave birth and said she does not know the reason why it started during this period. But she later explained that going outside in the sun putting on butter resulted in ‘likift’ (spirit attack): Participant: I went outside in the sun while I was putting on butter when I was አራስ [a woman who has recently delivered a baby]. My mouth and my body became swollen. My body stopped working, I could not eat, I could not go outside, I could not sleep. Then my sister who lives in Addis Ababa came and took me to the holy water place (Patient 4, female) Interviewer: What do you think is the cause of your illness? Participant: The illness eh God brings it to me what else do I think eh it comes from እግዚያብሄር እንዳመጣብኝ ነው እንጂ እኔ ምንም አስባለሁ …እ… በምን እንደመጣ ነው Interviewer: Do you know the reason? Participant: I don't know the reason (Patient 5).

Explanations related to cultural practices	Dried body and scalp because of not putting on butter during the grief period Applying butter on body and hair is a common practice in Ethiopia.	Interviewer: How long it has been since this feeling began? Participant: It was a long time ago. It has been eight years. Interviewer: Eight years? Participant: Eight years. My father died and my head became like this as a result (አባቴ ሞቶ በዛ የተነሳ ነው ጭንቅላቴን እንትን ያለው) Interviewer: After the death of your father? Participant: Yes, after the death of my father my head started to be disturbed (ጭንቅላቴ እንትን ማለት መረባበሽ). It was assumed it is grief (ሀዘን). Then my mother died after two years. Then my brother died. Because of that, it was assumed it is grief (ሀዘን), it is because my body was dried. That is how this illness has become worsened (ሰውነትሽ ስለደረቀ ነው ስባል ስባል ነው ይሄ በሽታ እንትን ያለው የባሰው). Interviewer: Your body? Participant: Yes Interviewer: What does it mean your body is dried? Participant: Eh butter, in our country we put butter (ቅቤ እንቀባለን), yes [during] grief we cut our hair, eh yes for the sake of the dead we cut [our hair], we put on black clothes, eh we don't put on butter or oil [during bereavement period] so it was said that it is because of that your body has become dried and your hair has become dried (በሱ የተነሳ ነው በቃ ሰውነትሽ ስለደቀ ነው ፀጉርሽ እንትን ስለደረቀ ነው ሲሉ ያያ ስል ነው) (Patient 2).
HIV	Community stigmatization Not being able to take care of oneself; to be dependent on others because of ill health	A female participant who lives with HIV explained her distress in terms of not being able to do things independently as well as the community stigma she experienced: Interviewer: In your opinion what do you think is this problem? Participant: I have told you at the beginning. I think it is because of their stigmatization. First, they stigmatized me. They isolated me. They made neighbors not to enter [into my house]. They made people not to approach me. They even made people not to fetch water for me … people even used to change their street when they see me (Patient 24). This participant also expressed her distress in terms of not being able to do things independently.
Major traumatic event	Abduction	One of the female participants said her illness worsened after she was abducted and experienced traumatic experiences during the abduction process, including being beaten which resulted in broken legs and being forced to sleep in a manger so that her relatives would not trace her. She also noted that the perpetrators of the abduction were not charged and were freely living their life, which affected her health greatly. Marriage by abduction is a practice in which a man (often with the help of his friends) abducts the girl or woman he wants to marry. It may involve rape. Although Ethiopia criminalized such practice, it is still prevalent in many regions of Ethiopia. “It is after them [the abductors] that my stomach become sick (ከእነሱ በኃላ ነው እንዲያውም ጨጓራ በሽታ ሆንኩኝ), this all happened to me. It is after this I become unable to control myself” (Patient 13).
Did not know the cause of their illness		About a quarter of the participants (7/28) said they did not know the cause of their illness.
